# Masking as Myelofibrosis: A Case of Tp53 Mutated Acute Erythroid Leukemia Presenting With Pancytopenia and Bone Pain

**DOI:** 10.1155/crh/5864392

**Published:** 2025-11-04

**Authors:** Karsten Parker, R. Clark Cutrer

**Affiliations:** ^1^The University of Tennessee Health Science Center College of Medicine, Memphis, Tennessee, USA; ^2^The University of Tennessee Medical Center, Knoxville, Tennessee, USA

## Abstract

Acute erythroid leukemia is a rare form of acute myeloid leukemia, comprising only 1% of myelogenous leukemia diagnoses. Presentations can vary and given its aggressive nature, prompt investigation and appropriate treatment are needed when suspicions arise. Here, we discuss a case of a 54-year-old male who initially presented with worsening fatigue and dyspnea on exertion and was found to have significant pancytopenia. Bone marrow biopsy initially demonstrated significant fibrosis concerning for primary myelofibrosis, though JAK2 testing was negative. He was started on JAK inhibitor therapy with pacritinib but clinically declined over the next several days with worsening diffuse pain and pancytopenia. A repeat bone marrow biopsy demonstrated acute erythroid leukemia with biallelic Tp53 mutations. He was subsequently started on FLAG-Ida-Ven induction, with complete remission obtained after induction. Transplant work-up was started, and he received a cycle of FLAG-Ida-Ven consolidation. Shortly after, the patient presented to an outside hospital with septic shock, at which point the patient expired. This case illustrates the aggressive nature of the disease, the need for confirmatory testing when diagnosis is suspected and the difficulty in management as the prognosis is poor and requires aggressive treatment that can lead to life-threatening sequelae.

## 1. Introduction

Acute erythroid leukemia (AEL) is a rare subtype of acute myeloid leukemia (AML), with de novo mutations being the leading cause of AEL and accounting for less than 1% of de novo AML cases [1]. The fifth edition of World Health Organization (WHO) hematolymphoid tumors requires greater than 80% of bone marrow elements to have erythroid predominance, of which greater than 30% of these erythroid elements are proerythroblasts [2]. This case shows an interesting workup of a man presenting with constitutional symptoms and pancytopenia. He was initially diagnosed with myelofibrosis through bone marrow biopsy sampling. Subsequent bone marrow biopsies revealed the final diagnosis: AEL.

## 2. Case Description

The patient was a 54-year-old male that presented to University of Tennessee Medical Center (UTMC) after referral from his primary care provider following pancytopenia seen on complete blood count (CBC). The patient had been experiencing at least 2 months of unintentional weight loss, fatigue, night sweats and shortness of breath before presentation to the emergency department. The patient had no prior history of these symptoms. The patient self-reported a CBC performed in January of 2024 showing blood counts within normal limits. The patient's admission physical exam was remarkable for skin pallor. Admission CBC was remarkable for 6.1 g/dL hemoglobin, 16.9% hematocrit, 2 k/μL white blood cell count and 3 k/μL platelets. Two days after admission, a left iliac crest bone marrow biopsy was performed. Bone marrow biopsy resulted in an inadequate sample for full testing but suggested primary myelofibrosis. The patient followed up with the outpatient oncology clinic. He was placed on 200 mg pacritinib PO twice daily but continued to clinically regress with worsening fatigue, weight loss, low back and abdominal pain. After multiple biopsies and an outside hematopathologist read, the patient was diagnosed with AEL, with molecular testing showing two Tp53 mutations with a small variant allele frequency (VAF). The first Tp52 mutation was a p.R273H missense mutation with a VAF of 10.3%. The second Tp53 mutation was a p.Y234C missense mutation with a VAF of 12.9%. The patient was negative for CALR, FLT3, IDH1, IDH2, JAK2, MPL, and NPM1 mutations. The karyotype was normal but FISH was notable for gain of 19q13.3 and loss of 19p13.2, del 5q31 and gain of 11q.

Patient received induction chemotherapy with a fludarabine, cytarabine, idarubicin, and venetoclax-based treatment regimen with granulocyte colony stimulating factor support (FLAG-Ida-Ven). On day 21 of the protocol, the patient received a bone marrow biopsy showing hypocellular bone marrow with no active disease. The patient was later discharged on day 34 of induction with multiple infectious complications and referred to the UT transplant center for consideration of allogeneic bone marrow transplant. He was shortly after readmitted for cycle one of consolidation with FLAG-Ida-Ven and discharged after 5 days. However, sadly, while awaiting count recovery and a hematopoietic stem cell transplant, the patient presented to an outside hospital 8 days later with severe shortness of breath, was found to be septic with pancytopenia, and ultimately expired in the emergency department after expressing a desire for a do-not-resuscitate code status.

## 3. Discussion

This unfortunate case provides new insights into an exceedingly rare disease: AEL. AEL is a myeloid neoplasm that accounts for 1% of de novo AML cases. With an incidence of 21,450 AML cases in the United States in 2019, this would suggest roughly 200 cases of AEL are diagnosed each year per the 2016 WHO leukemia guideline classification update [3].

2016 and 2022 WHO classification changes have made AEL a less frequent diagnosis. It becomes more difficult to retrospectively identify cases of AEL, since the currently restrictive criteria disqualify many previous cases of AEL. The prognosis of AEL is generally considered poor, although recent classification changes make this difficult to quantify. Liu et al. show through their literature survey that remission rates of aberrant versus normal karyotype AEL are 37% and 83%, respectively [4]. This study was published in 2006, again calling into question the exact quantification of survival statistics for the most recent WHO criteria.

It was unclear if this was a secondary AML from previously existing myelofibrosis or a missed diagnosis of AEL upon initial bone marrow biopsy due to presence of fibrosis and lack of specific erythroblastic staining done initially.

AML treated with the FLAG-Ida induction chemotherapy protocol shows improved overall survival (OS) compared to the “7 + 3” daunorubicin and cytarabine induction regimen, but composite complete remission (CRc) remains relatively low at 57% with the FLAG-Ida induction in newly diagnosed acute myelogenous leukemia (ND-AML) patients [5]. A 2022 literature survey of younger patients (median age of 44 years with a range from 20 to 65 years) with no comorbid conditions and ND-AML found that the FLAG-Ida induction protocol benefits from the addition of a BH-3 mimetic, such as venetoclax [6]. The CRc improves to 89% with the addition of venetoclax to the FLAG-Ida induction protocol [6]. Induction chemotherapy in patients with double allelic Tp53 mutations is less well studied, although 2-year OS remains poor when compared to wild-type Tp53 alleles regardless of treatment intensity [7]. Recent studies suggest improved outcomes with intensive chemotherapies for mut-Tp53 patients such as FLAG-Ida-Ven are more effective than less intensive therapies [8]. This case provides additional evidence that induction therapy for AEL should include the addition of venetoclax in the FLAG-Ida regimen for young patients with no comorbid conditions.

## Data Availability

The data that support the findings of this study are available on request from the corresponding author. The data are not publicly available due to privacy or ethical restrictions.
